# Comparison of cytotoxicity of Miltefosine and its niosomal form on chick embryo model

**DOI:** 10.1038/s41598-024-52620-4

**Published:** 2024-01-30

**Authors:** Fatemeh Seyedi, Iraj Sharifi, Ahmad Khosravi, Elaheh Molaakbari, Hadi Tavakkoli, Ehsan Salarkia, Sina Bahraminejad, Mehdi Bamorovat, Shahriar Dabiri, Zohreh Salari, Ali Kamali, Guogang Ren

**Affiliations:** 1 Department of Anatomy, School of Medicine, Jiroft University of Medical Sciences, Jiroft, Iran; 2 Leishmaniasis Research Center, Kerman University of Medical Science, Kerman, Iran; 3 Department of Chemistry, Shahid Bahonar University of Kerman, Kerman, Iran; 4 Department of Clinical Science, School of Veterinary Medicine, Shahid Bahonar University of Kerman, Kerman, Iran; 5 Afzalipour School of Medicine and Pathology and Stem Cells Research Center, Kerman University of Medical Sciences, Kerman, Iran; 6 Obstetrics and Gynecology Center, Afzalipour School of Medicine, Kerman University of Medical Sciences, Kerman, Iran; 7 Department of Infectious Diseases, School of Medicine, Jiroft University of Medical Sciences, Jiroft, Iran; 8 School of Engineering and Computer Science, University of Hertfordshire, Hatfield, AL10 9AB UK

**Keywords:** Cell biology, Developmental biology

## Abstract

Various drugs have been used for the treatment of leishmaniasis, but they often have adverse effects on the body's organs. In this study, we aimed to explore the effects of one type of drug, Miltefosine (MIL), and its analogue or modifier, liposomal Miltefosine (NMIL), on several fetal organs using both in silico analysis and practical tests on chicken embryos. Our in silico approach involved predicting the affinities of MIL and NMIL to critical proteins involved in leishmaniasis, including Vascular Endothelial Growth Factor A (VEGF-A), the Kinase insert domain receptor (KDR1), and apoptotic-regulator proteins (Bcl-2-associate). We then validated and supported these predictions through in vivo investigations, analyzing gene expression and pathological changes in angiogenesis and apoptotic mediators in MIL- and NMIL-treated chicken embryos. The results showed that NMIL had a more effective action towards VEGF-A and KDR1 in leishmaniasis, making it a better candidate for potential operative treatment during pregnancy than MIL alone. In vivo, studies also showed that chicken embryos under MIL treatment displayed less vascular mass and more degenerative and apoptotic changes than those treated with NMIL. These results suggest that NMIL could be a better treatment option for leishmaniasis during pregnancy.

## Introduction

Leishmaniasis is a disease that poses a significant public health challenge. It is caused by an intracellular parasite belonging to the Leishmania genus and is typically transmitted to humans through the bites of infected phlebotomine sandflies when they feed on blood^1–3^.

Leishmaniasis can present in different ways and may cause various clinical symptoms. Some forms of the disease, such as visceral leishmaniasis (VL) and cutaneous leishmaniasis (CL), are of particular concern^4^. Leishmaniasis is a disease widely found in tropical and subtropical regions, including North Africa, Asia, and the Middle East. Each year, it affects approximately 1.5–2 million people worldwide and is caused by more than 20 species of Leishmania parasites. Additionally, over 1 billion people globally are at risk of contracting leishmaniasis due to factors such as poverty, malnutrition, weakened immune systems, and proximity to breeding sites of sandflies, which are the vectors for the disease^5,6^.

Miltefosine (MIL), also known as hexadecylphosphocholine, is an oral medication for leishmaniasis. The recommended dose for MIL is 2.5 mg/kg/day for four weeks. Clinical trials in India have reported a cure rate of up to 98% for patients with visceral leishmaniasis (VL) treated with MIL^7^.

In previous studies, it has been determined that the mechanism of action of MIL is by decreasing oxygen consumption and reducing the level of ATP through the inhibition of mitochondrial cytochrome c oxidase. Also, an increase in apoptosis-like death in promastigotes of different Leishmania species has been observed^8^.

However, various studies have revealed that MIL use is associated with frequent side effects. Nevertheless, MIL is effective and safe in pediatric patients with leishmaniasis^9,10^. In a previous study, the toxicity of MIL was evaluated in rats during organogenesis and early embryonic development. The study found that even at lower clinical doses (1–2 mg/kg), MIL caused fetotoxic, embryotoxic, and teratogenic effects in the rats. Similar results (excluding teratogenic effects) were obtained for rabbits during organogenesis. As a result, MIL has been classified as a contraindicated drug in pregnancy and assigned to category D by the United States Food and Drug Administration (FDA)^10^. While many clinical trials have been published on MIL's efficacy in treating patients with VL and CL, studies assessing the side effects of MIL treatment in pregnant women are rare.

Although the incidence of VL during pregnancy is uncommon worldwide, it can still pose a threat to the life of the mother and have irreparable effects on the fetus. If treatment of pregnant women fails, VL can cause vertical transmission and fetal death. Moreover, current chemotherapies have the potential for teratogenic consequences and toxicity to the fetus. Therefore, it is essential to develop new strategies for treating leishmaniasis during pregnancy^11–13^. Niosomes are a promising drug delivery system that could be used to treat leishmaniasis patients. They are a type of vesicular system that offers several advantages, such as the ability to encapsulate both hydrophilic and hydrophobic drugs, high levels of uptake by the reticuloendothelial system, and low toxicity, thus allowing for targeted and sustained drug delivery^14–16^.

This study investigates the effects of MIL, one of the effective oral drugs used in leishmaniasis treatment, on the processes of apoptosis and angiogenesis. This was achieved through molecular docking of a chick embryo model as an in vivo assay and in vitro models on the chicken embryo.

## Methods

### Noisome preparation

The drug MIL was procured from the company Sigma (CAS-Number: 58066-85-6), and the original niosome and NMIL were prepared using the thin layer evaporation method. This method was chosen to enhance the drug's bioavailability and efficacy^17,18^. The three ionic surfactants used to prepare niosomes were Span 60, Tween 60, and cholesterol^19,20^. To prepare either the unloaded niosomes or the NMIL dispersion, chloroform was used to dissolve a mixture of Span 60, Tween 60, and cholesterol (300 μmol). The solution was transformed into vapor using a rotary evaporator under 20 inches of mercury vacuum at 60 °C and 120 rpm. This process resulted in a uniform and dry lipid thin film. The thin film was then soaked in a solution of MIL (1.72 × 10–3 M in PBS pH 7.4) to obtain the NMIL dispersion. The soaking was carried out at 60 °C and 160 rpm for 60 min, after which the niosomal suspension was allowed to equilibrate at room temperature for 12 h. This step was necessary to distribute the MIL between the phospholipid bilayer and the liquid phase, leading to an optimal NMIL dispersion^20^.

For a uniform size distribution of niosomes containing MIL, it is essential to utilize a sonicator probe with an amplitude of 100 W and sonicate the sample for at least 15 min while maintaining it in an ice bath. Once done, filtering the resulting small unilamellar vesicles (SUVs) through 0.44 µm and 0.22 µm membranes is crucial to attain the desired outcome.

#### Characterization of niosomes

The classification parameters of niosomes significantly impact their stability and can provide essential information on their in vivo performance. Therefore, it is crucial to evaluate several characteristics, such as morphology, zeta potential, size, encapsulation stability, and efficiency, to ensure the effectiveness of niosomal formulations. To determine the construction and investigate the chemical attachment types between MIL and the niosome machinery (span 60, tween 60, and cholesterol), the identification of samples' functional groups was investigated using a Fourier transform infrared (FTIR) spectrometer. The FTIR spectrum was obtained by analyzing the sample within a wavelength range of 400–4000 cm^−1^.

#### Size and morphology

The techniques widely used to determine the size and morphology of niosomes are dynamic light scattering (DLS), transmission electron microscopy (TEM), and scanning electron microscopy (SEM)^21,22^. DLS is a technique that allows for the simultaneous collection of relevant information on particle size and its uniformity in suspension. In the DLS contour, a single sharp peak indicates the presence of a single population of scatterers. SEM and TEM are imaging techniques that provide high-resolution images of the surface and interior structure of niosomes, respectively.

#### Zeta potential

Utilizing the Zeta-sizer instrument was essential in determining the size and zeta potential of the niosomes. Notably, the surface charge of these niosomes is a vital characteristic that can substantially impact their overall performance. Generally, uncharged niosomes are less stable against aggregation than charged ones. The physicochemical properties of niosomes, such as the negative zeta potential value, indicate that the electrostatic balance of niosomes is satisfactory for a balanced suspension system^23^. The polydispersity index (PDI) of less than 0.4 indicates a homogeneous population for colloidal systems^22^.

#### Entrapment efficiency

Parts of the drug (or %) trapped within or by niosomes are defined as Entrapment Efficiency (EE%). The free drug was settled using centrifugation of the noisome suspension. Furthermore, by vesicle destruction, the capsulated drug was released from niosomes. This was performed by adding additional methanol to the niosomal solution. The concentration of the loaded drug and the free drug was ascertained by a UV spectrophotometer with absorption at wavelength 269 nm^24^ where Ci is the initial concentration of the medication utilized to make biosomes, and Cf signifies the attention of the compound without entrapment.

#### Stability

The niosomes’ stability was evaluated by defining the mean of the vesicle size, size distribution and the efficiency of entrapment beyond several-month storage periods. Samples were checked in orderly distance time under UV spectroscopy to find the percentage of drug storage in noisome^24,25^.

#### In vitro release

The method employed to investigate the in vitro release was by applying a dialysis bag that was splashed and immersed in double distilled water. The drug-loaded niosomal solution was transported to the tubing. The dialysis bag encompassing niosomes was deep in PBS solution, stirring at 37 °C. In determining time duration, samples were removed from the outer buffer and exchanged with the same amount of washing buffer. The models were analyzed toward the drug contented by a UV spectrometer scheme^26,27^.

### In silico study

#### Ligand preparation

**Figure 1:**
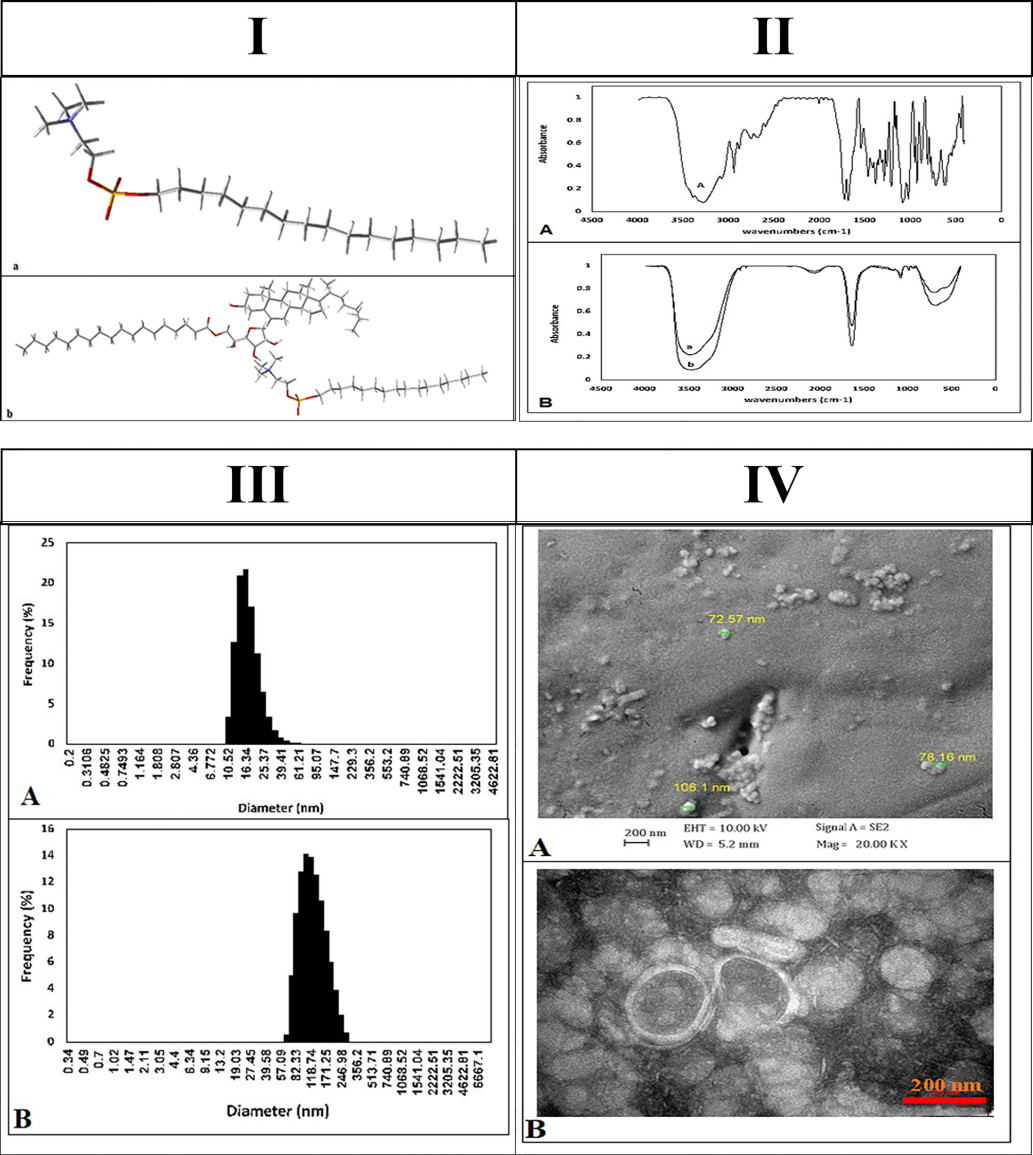
(**I**) Conformer of (**a**) MIL and (**b**) NMIL structures used in docking process. (**II**) FTIR spectra of (**A**) free Miltefosine, (**B**) niosomal Miltefosine (**III**) Gaussian size distribution for (**A**) unloaded niosome and (**B**) niosomal Miltefosine (**IV**) (**A**) SEM, and (**B**) TEM image of niosomal Miltefosine.

To design the NMIL structure, the Span 60, Tween 60, cholesterol, and MIL conformers were obtained from PubChem compounds available through the National Center for Biotechnology Information (NCBI) database and downloaded in SDF format. The niosome structure was designed based on the article by Ritwiset et al.^28^. The geometries of the niosome compound were optimized using HyperChem software. In the optimization stage, the molecular mechanics force field of MM + was employed, followed by the semi-empirical method of AM1 to improve the structures. Final structures were saved in Mol2 format. Molecular quantum designs of NMIL were carried out using the DMol3 module and Materials Studio software^29^. Energy advance and geometry configuration, including HOMO and LUMO energy levels of the components, was analyzed by the DMol3 component. Energy escalation, HOMO and LUMO computations were carried out, and a combination of both molecules (MIL and niosome) was employed to estimate configurations of the energy and the mixture distribution. The preferable structure with the most stable NMIL position and lowest energy was selected and stored in SDF format. These selected ligand structures were used as input for the molecular docking software. Figure 1(I) shows the chosen ligand structures built using the proper input for molecular docking software.

#### Target/receptor preparation

The text describes a study focused on the molecular docking of several proteins involved in angiogenesis regulation and apoptosis control. The researchers downloaded the structures of the target proteins, including Bcl-2, BAX, VEGF-A, and Caspase-8 receptors, from the RCSB protein database (https://www.rcsb.org/) using their corresponding PDB IDs. They then used MVD software to remove any additional factors in the PDB heading and construct models for molecular docking.

#### Molecular docking (MVD) process

We utilized MVD software to sort out the protein structures and combinations and identify the suitable openings on the receptors for binding with ligands. We set up a docking framework with a grid strength of 0.30 Å, a maximum iteration of 1500, and a maximum size of 50. To evaluate the necessary affinity with BAX, Bcl-2, Caspase-8, and VEGF-A, we recorded the internal electrostatic interactions, sp2-sp2 rotations, and internal hydrogen bond connections. We employed the Simplex progress method with 300 high ladders and a neighborhood remoteness factor of 1. After running ten sets of docking, we conducted post-dock energy minimization through Nelder-Mead Simplex Minimization. The Molegro Molecular Viewer and Discovery Studio were used to evaluate the products, and we chose the best-related composite from each dataset. The precision of the docking was assessed, with AutoDock's Vina package achieving approximately 78% precision, higher than AutoDock 4.2^30^, while MVD software achieved a precision accuracy of close to 87%^31–34^.

#### ADME and toxicity estimates

Access AdmetSAR through this link: http://lmmd.ecust.edu.cn/admetsar2/. Utilizing this valuable and free resource is imperative for predicting the ADMET properties of new chemical compounds. AdmetSAR is a drug detection server that employs computational methods to evaluate compounds' ADME and toxicity properties^35,36^.

### In vivo examination

For validation of the anti-angiogenic properties of MIL through analysis of the embryo using the yolk sac membrane (YSM) assay (all the study processes have been completed until embryonic day 7). In molecular assays, changes in anti-apoptotic (TP53T Apaf1, Bax and Bcl-2) and anti-angiogenic (VEGF- KDR) gene expression were analyzed by qRT-PCR method. At first, the total RNA of YSM vasculature was extracted by triazole (YTzol Pure RNA, Iran), and cDNA was synthesized by using the TaKaRa kit, finally, by using the specific primers and reference gene sequences listed in Table 1. The qPCR reaction was done using Parstous RTSYBR Green in Rotor-Gene Q^37,38^.

**Table 1:** List of primers.

Template	Forward primer	Reverse primer
TP53	TCCTCTTGGGCATGACTACC	TGTCAATCTTGCTGCTCACC
Apaf1	TTGCCAACCAAGACATCAGAGG	TGCGGACGAACAACCAGACG
Bax	CCCGAGAGGTCTTTTTCCGAG	CCAGCCCATGATGGTTCTGAT
Bcl2	AGCGTCAACCGGGAGATGT	GCATCCCATCCTCCGTTGT
VEGF	CAATTGAGACCCTGGTGGAC	TCTCATCAGAGGCACACAGG
KDR1	GGAGTTTCCCAGAGACCGAC	CAATCCCAAAGGCATCAGC
HPRT	GATGAACAAGGTTACGACCTGGA	TATAGCCACCCTTGAGTACACAGAG
GAPDH	CCTCTCTGGCAAAGTCCAAG	GGTCACGCTGGAAGATA

The specific primers and reference gene sequences for quantitative real-time RT-PCR.

Investigating histopathological changes by observing slides stained with hematoxylin and eosin (H&E) under a light microscope, as well as investigating immunohistochemical (IHC) changes in angiogenesis(CD34) and apoptosis (Bax and Bcl2) markers.

#### Statistical analysis

SPSS and GraphPad Prism software were used for statistical analysis of the results of this study using an independent sample t-test and one-way ANOVA. The significance limit was considered *P* ≤ 0.05.

#### Ethical standards

The present study was approved by the Animal Administration and Ethics Committee of Jiroft University of Medical Sciences Sciences (Approval ID. IR.JMU.REC.1398.032).

## Results

### Drug preparation

#### Morphological characterization of unloaded niosome and NMIL

FTIR spectroscopy studies were performed to investigate the chemical composition of the niosomes. Figures 1(II) A-B provide spectra from free MIL and a comparison between spectra from unloaded niosomes and NMIL. The strong peak between 2800 and 3000 cm^−1^ is associated with the C–H extension. The band around 1643 cm^−1^ is likely from the carbonyl group (C=O). Additionally, the peaks in 1087 cm^−1^ and below 1000 cm^−1^ indicate –C–CO–O– and aliphatic C–H, indicating the presence of the surfactant. However, considerable overlapping in the spectra of unloaded and NMIL was observed, indicating that the chemical behavior of the niosomes remained unchanged. The FTIR study provides evidence, that no chemical interactions between the MIL and the nanostructures may have altered its chemical structure or composition throughout the preparation and processing and that the MIL was physically encapsulated in the niosomes.

The mean diameters of both unloaded and MIL-loaded niosomal constructions, consistent PDI, MIL entrapment efficiency (EE%) values, and zeta potential values are presented in Table 2. Gaussian size distribution of the unloaded and MIL-loaded niosomes obtained from their DLS measurement is shown in Figs. 1(III) A and B. The size range of the unloaded vesicles was measured to be between 36 and 151.0 nm. Interestingly, after MIL loading, the hydrodynamic diameter of the vesicles increased to 50–300 nm.

**Table 2:** Characterization of unloaded niosome and niosomal Miltefosine.

Characterization parameter	Zeta potential (mV)	Polydispersity index (PDI)	Z-average (r.nm)	Entrapment efficiency (%)
Unloaded niosome	− 27.3	0.377	49.76	–
Niosomal Miltefosine	− 15.7	0.163	119.5	94.4

The surface charge of nanoparticles significantly impacts cellular interactions and nanoparticle uptake. Studies have suggested that nanoparticles with negative or positive charges are more easily internalized than their uncharged counterparts^39^. However, nanoparticles with positive charges have been shown to promote rapid cellular uptake, while negatively charged nanoparticles were found to induce hemolytic effects on normal tissues^39,40^. The synthesized unloaded and NMIL niosomes showed a zeta potential of − 27.3 and − 15.7, respectively, indicating the stability of the colloidal suspension of the niosomes. The coulombic repulsion forces resulting from the charge on their surface overcome the van der Waals attractive forces.

Surface imaging of the niosomes was conducted using SEM. The SEM image shown in Fig. 1(IV)A illustrates NMIL at 200 nm, which has an even, sphere-shaped morphology with flat surfaces. The size of the NMIL was around 80–150 nm. Figure 1(IV)B shows the TEM image of NMIL, which illustrates the round configuration and robust structure of the niosomes. The TEM image further confirms that the NMIL are in deep vesicular forms with a diameter of less than 150 nm, consistent with the final product configuration.

#### Drug entrapment

**Figure 2:**
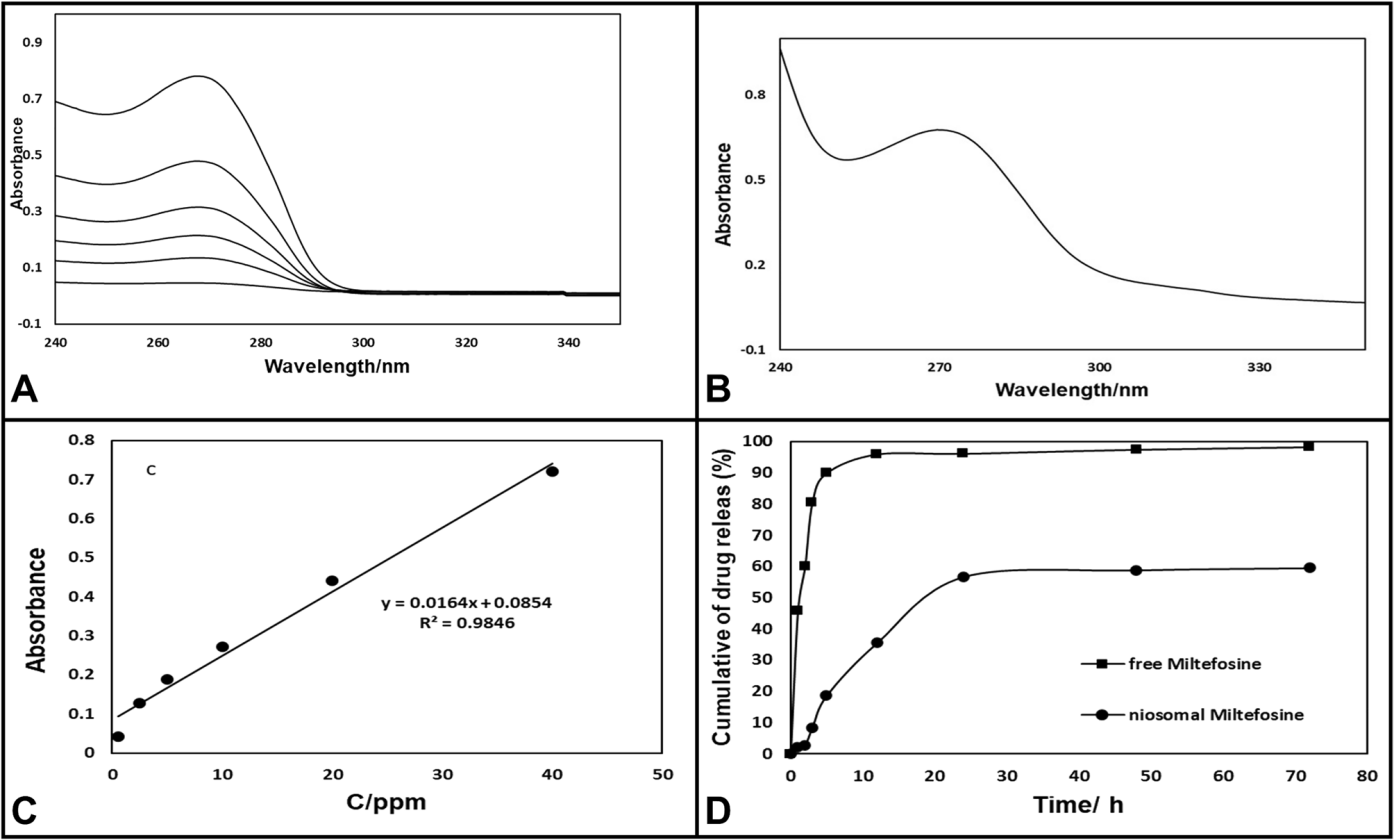
Diagram of UV–Vis absorption spectroscopy of (**A**) 2.5, 5, 10, 20, 40 ppm of MIL (MIL) (**B**) 30 ppm of NMIL. (**C**) Typical standardization curve of the MIL. (**D**) In vitro drug release profile of MIL.

Confirmation of entrapment efficiency was established using UV–Vis absorption spectroscopy. Figure 2A depicts the absorbance of niosomal Miltefosine in comparison with unloaded niosome and free Miltefosine. Miltefosine (free drug) shows l max at wavelength 269 nm, where a significant peak was observed, as shown in (Fig. 2B). The Same peak was also observed in the case of niosomal Miltefosine, depicting Miltefosine entrapment in niosomes. However, the peak was not as prominent as observed in the case of free drug, which may be due to the small amount of drug entrapment inside the niosomal nanocarrier. The minor peak shifts observed could be due to the interaction of Miltefosine with niosome constituents during the synthesis and encapsulation processes. In a matter of unloaded niosome, no peak was observed. As a result, the calibration curve of miltefosine was depicted using the UV curve, as shown in Fig. 2C. Also, The entrapment efficiency of the particles was 94.37% for every batch of particles synthesized in the drug getting encapsulated, as seen in Table 2.

#### In vitro drug release

In vitro drug release Evaluation was accomplished by utilizing the dialysis bag approach and the results of the release profile for free Miltefosine and niosomal form (in PBS pH 7.4 at 37 °C) were provided in Fig. 2D. The release of Miltefosine in niosomal form was considerably fewer than Free Miltefosine. Unloaded Miltefosine demonstrated a rapid release diagram with a release acceleration of approximately 85% in 4 h and is about constant followed (within 48 h, 97.5% of the drug is released). However, the release profile of Miltefosine in niosomal form in Fig. 2D offered the loaded drug was released after 48 h, 58.6%. Consequently, the influential role of niosomal formulations in modifying expeditious release at physiological (pH 7.4) is noticeable.

### In silico study

#### MVD molecular docking

**Figure 3:**
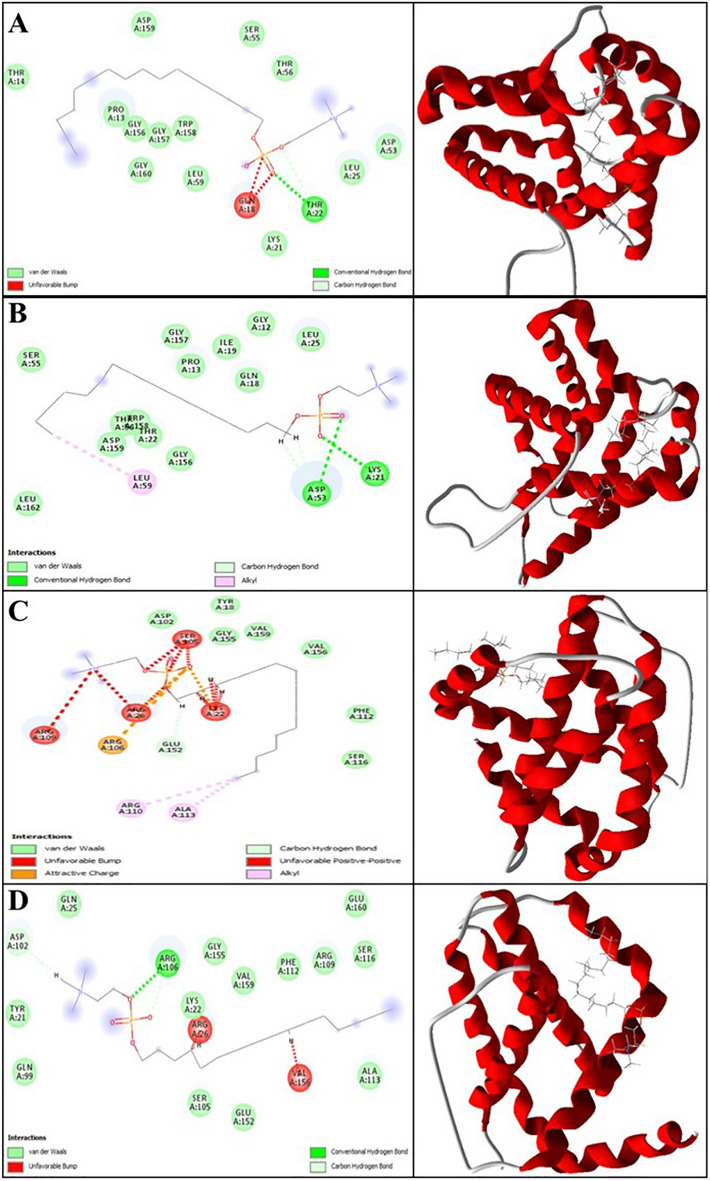
Representation of the best score docking solution of the MIL ligands and (**A**) BAX and (**C**) Bcl-2 receptor and NMIL and (**B**) BAX and (**D**)Bcl-2 receptor with the designated crystal structure of 5W5X and 5JSN, respectively, along with the ligand map with various chemical bonds of Discovery Studio.

**Figure 4:**
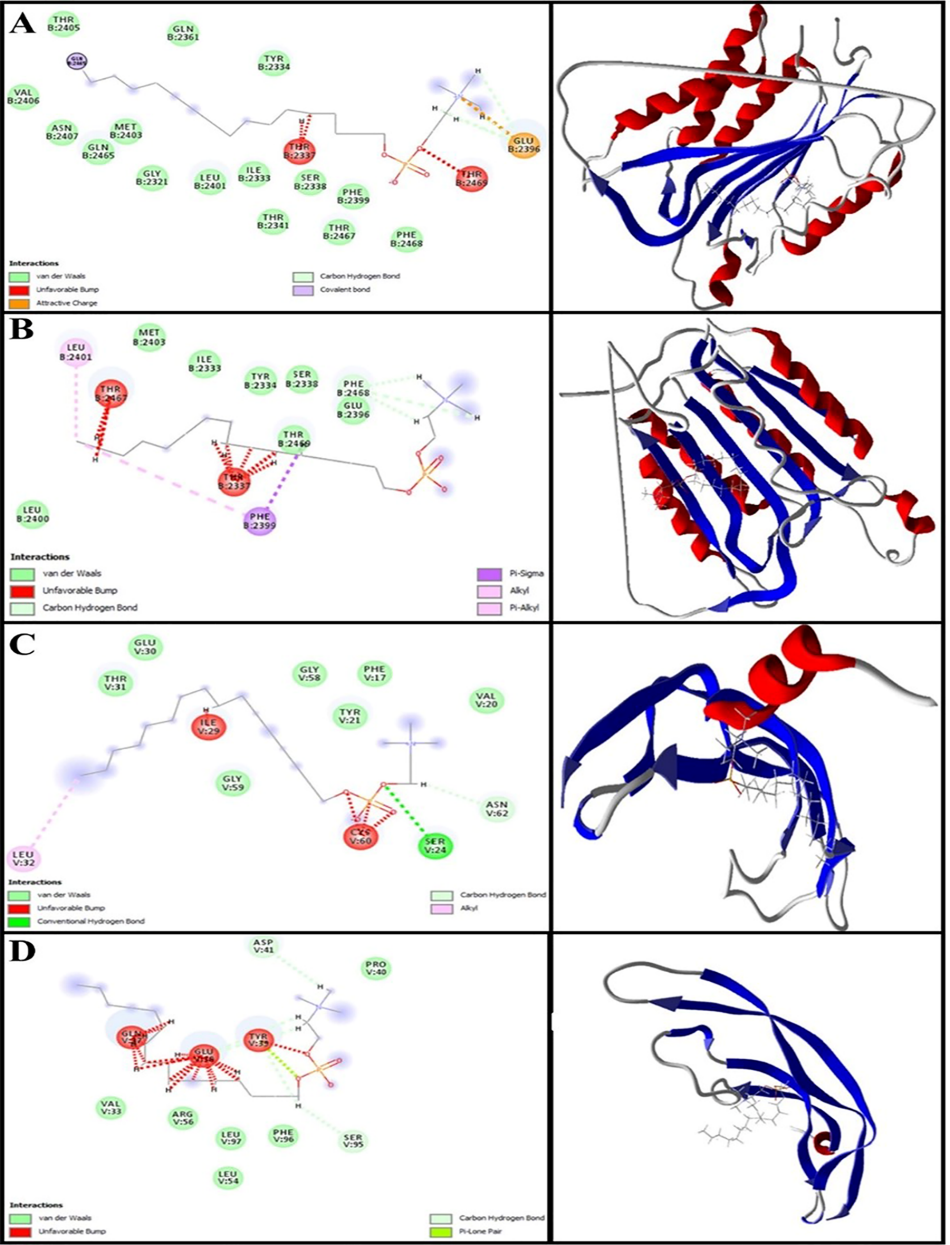
Representation of the best score docking solution of the MIL ligands and (**A**) Caspase-8 and (**C**) VEGF-A receptor and NMIL and (**B**) Caspase-8 and (**D**) VEGF-A receptor with the designated crystal structure of 1I4E and 5T89, respectively, along with the ligand map with various chemical bonds of Discovery Studio.

Using modeling packages such as BAX, Bcl-2, Caspase-8, and VEGF-A, an in silico investigation was conducted into the conformation of docked MIL and NMIL. The analysis focused on their energy levels, precisely the Free Total Energy and MolDock Score values, which yielded negative energy values. These findings unequivocally demonstrate that the binding events between the composites occurred spontaneously. This research focuses on the interactions between the free drugs and drugs in NMIL. Therefore, only drug interaction was studied after molecular docking of the drug in the NMIL form. The conformity of the mixtures with their respective parameters is shown in Table 3. MIL achieved MolDock Score values of − 77.27, − 103.924, − 111.623, and − 80.8551 when docked to BAX, Bcl-2, Caspase-8, and VEGF-A receptors, respectively. On the other hand, NMIL achieved MolDock Score values of − 101.351, − 92.551, − 114.929, and − 78.4012 when docked to the same receptors. Figures 3 and 4 showcase the molecular docking results for targets 5W5X, 5JSN, 1I4E, and 5T89. The ligand maps were explored, revealing that MIL formed Conventional Hydrogen Bonds, Carbon Hydrogen Bonds, and Unfavorable Bumps with amino acid residues (Gln 18 and Thr 22) of the BAX receptor (Fig. 3A). On the other hand, NMIL formed van der Waals, conventional hydrogen bonds, Alkyl, and Carbon Hydrogen Bonds with amino acid residues of the BAX receptor, including Leu 59, Asp 53, and Lys 21 (Fig. 3B). It has been discovered that MIL binds to Bcl2 through a binding site that includes specific amino acid residues, such as Lys 22, Arg 26, Ser 105, Arg 106, Arg 109, and Glu 152. These residues have attractive charges from Alkyl, Carbon Hydrogen Bonds, and Unfavorable Bumps (Fig. 3C). Docking examination data (displayed in Fig. 3D) further reveals that NMIL forms van der Waals, Conventional Hydrogen Bonds, Carbon Hydrogen Bonds, and opposed bumps with amino acids of the Bcl2 using Arg 26, Asp 102, Arg 106, and Val 156.

**Table 3:** Resulted parameters from the interaction between the Miltefosine and Miltefosine in the niosomal form and BAX, Bcl-2, Caspase-8 and VEGF-A receptor.

Compound	Docking score for Miltefosine	Docking score for niosomal forms
BAX	− 77.27	− 101.351
Bcl-2	− 103.924	− 92.551
Caspase-8	− 111.623	− 114.929
VEGF-A	− 80.8551	− 78.4012

Regarding MIL's interaction with Caspase-8, the research found that it formed various bonds with amino acids utilizing Thr 2337, Glu 2396, Gln 2465, and Thr 2469. These included van der Waals, Covalent, Attractive Charge, Alkyl, Carbon Hydrogen, and Unfavorable Bumps (as shown in Fig. 4A). In addition, Caspase-8 stabilized NMIL through bonds with amino acid residues such as Thr 2337, Glu 2396, Phe 2399, Leu 2401, Thr 2467, and Phe 2468. These bonds included van der Waals, Alkyl, Carbon Hydrogen, Pi-Sigma, Pi-Alkyl, and Unfavorable Bumps (Fig. 4B). The apoptosis inherent pathway is initiated by interacting with Bcl-2 family proteins, including antiapoptotic proteins such as Bcl-2, pro-apoptotic proteins (BAX), and downstream mitochondrial indicators^41^. This cavity contains amino acids necessary for contact with the BAX carboxyl-terminal transmembrane area (Suzuki et al., 2000). Bcl-2 family proteins are essential membrane proteins, especially when included on the outer membrane of mitochondria, which play a vital role in regulating and conducting apoptosis^42^. According to this research, both MIL and NMIL affect the proteins related to cell death and survival, including BAX, Bcl-2, and Caspase-8. However, the relative free total energy of the NMIL and MIL with apoptotic-regulator proteins shows significantly increased interactions with BAX and Caspase-8 and decreased interactions with Bcl2 proteins. Consequently, NMIL was found to be more effective than MIL alone.

According to Fig. 4C, it is evident that the residues Glu 30 and Leu 32 of VEGF-A form interactions with NMIL through van der Waals, Conventional Hydrogen Bonds, and Carbon Hydrogen Bonds. Moreover, MIL is observed to attach to the active site of VEGF-A through van der Waals, Conventional Hydrogen Bonds, Carbon Hydrogen Bonds, and Unfavorable Bumps. The binding site essential for attachment comprises amino acid residues Ser 24, Ile 29, Leu 32, Cyc 60, and Asn 62 (as illustrated in Fig. 4D).VEGF-A is a significant agent for de novo blood vessel formation^43^. The results indicate that MIL's binding affinity was higher than that of NMIL with VEGF-A.

#### ADMET prediction

ADMET is commonly used before experimental procedures in prediction analysis related to chemical and biological processes. It helps in predicting the pharmacokinetics of molecules^44^. ADMET outcomes indicated that MIL had a lower Human Intestinal Absorption (HIA) score than NMIL, indicating that the NMIL complex may be less absorbed from the intestinal tract when administered orally. In contrast, NMIL was found to have the most significant penetration within the Blood–Brain Barrier (BBB). ADMET was also used to predict the outflow by P-glycoprotein (P-gp), but both complexes were found to be substrate inhibitors of P-gp.

Similarly, regarding metabolism, it was observed that NMIL was a CYP450 microsomal substrate enzymes (However, it is not an inhibitor). When a molecule is classified as a CYP450 non-inhibitor, it implies it won't hinder the bio-transformation process of drugs metabolized by the CYP450 enzyme. The AMES assay can test drug toxicity and is helpful in categorizing mutagenic compounds. Both NMIL and MIL were found to have negative AMES adverse events, indicating that they are not mutagenic. Moreover, the carcinogenic profile showed that the compounds were not carcinogenic. Our research has revealed that NMIL has lower oral toxicity than MIL. This prediction was made through ADMET analysis using the LD50 dose in a rat model. It's generally understood that a higher LD50 dose of a substance is less toxic than a lower LD50 dose. Our findings indicate that NMIL has a higher LD50 than MIL (2.9121 versus 2.9481, respectively), which means that MIL is more toxic than NMIL due to its lower LD50. Furthermore, it's worth noting that the solubility of a substance decreases with an increase in the log *S* value. This decrease in solubility would eventually lead to a lower absorption rate^45^. Consequently, it has been found that MIL with a lower log S value has superior absorption compared to NMIL, which indicates a higher level of bioavailability. Conversely, the higher log S value of NMIL suggests lower solubility and may result in lower absorption. However, this property may make NMIL more immune to hydrolysis and oxidation, improving drug stability and safety towards degradation. In turn, this could lead to increased bioavailability of NMIL compared to MIL molecules^46^.

### In vivo

#### Effect of MIL on the vascular density

**Figure 5:**
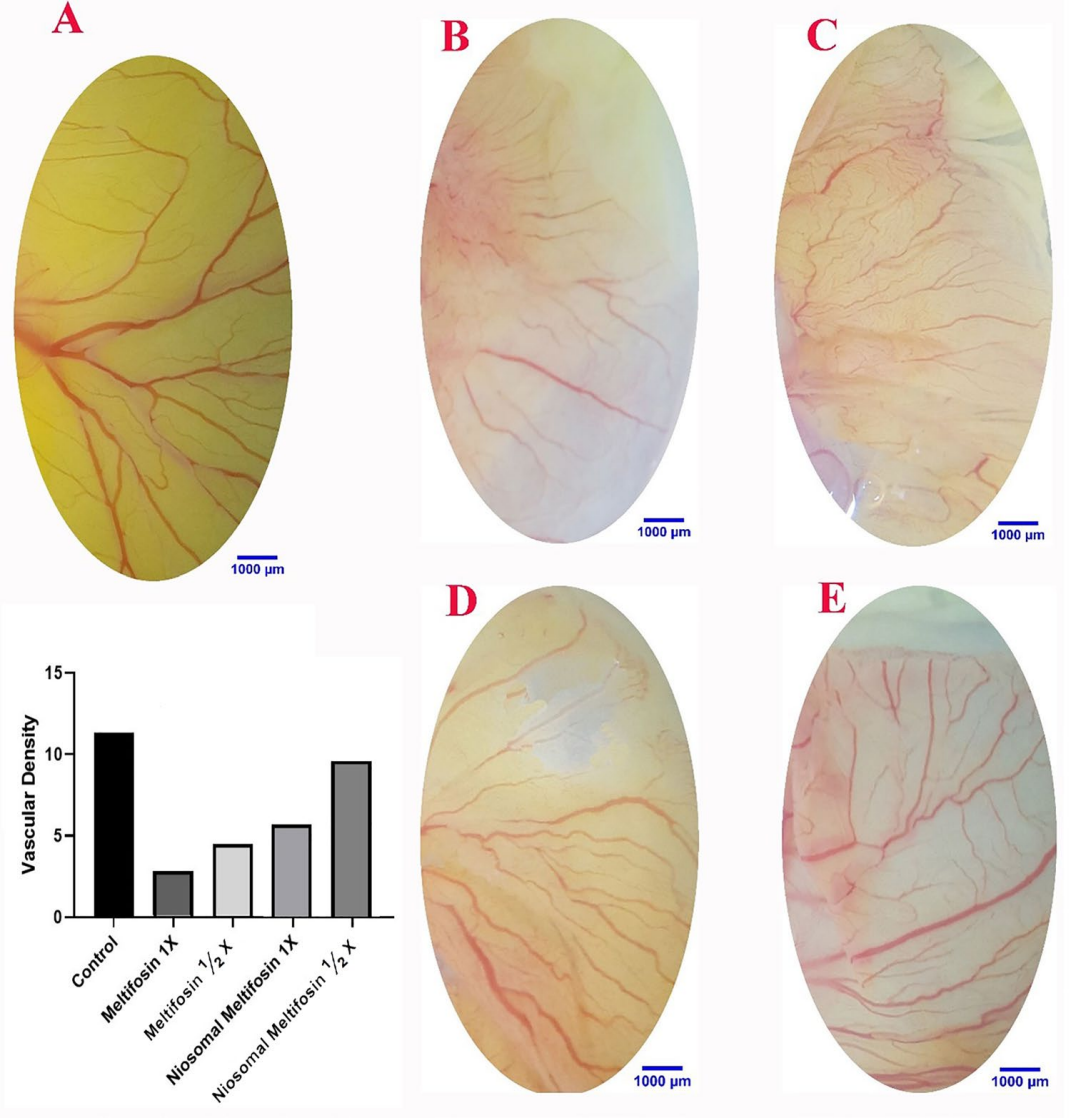
Effect of MIL and its NMIL within the blood vessel system. (**A**) Control embryo with characteristic blood vessel system. MIL (**A**) 1 × and (**C**) 2x, respectively, where the blood vessel system is interrupted. NMIL (**D**) 1X and (**E**) 2X, respectively, in contrast to MIL, which demonstrated a minor decrease in vascular mass. Vascular densities following treatments of MIL and its NMIL. A significant reduction in vascular density can be seen in both groups that received MIL and its NMIL. The embryos that received the MIL treatment revealed less vascular mass than NMIL.

The Yolk Sac Membrane (YSM) study showed that the growth of fetal vessels in the group receiving MIL was less compared to NMIL. Still, in both groups, a significant decrease in the development of fetal vessels was observed compared to the control group (Fig. 5).

#### Gene expression results

**Figure 6:**
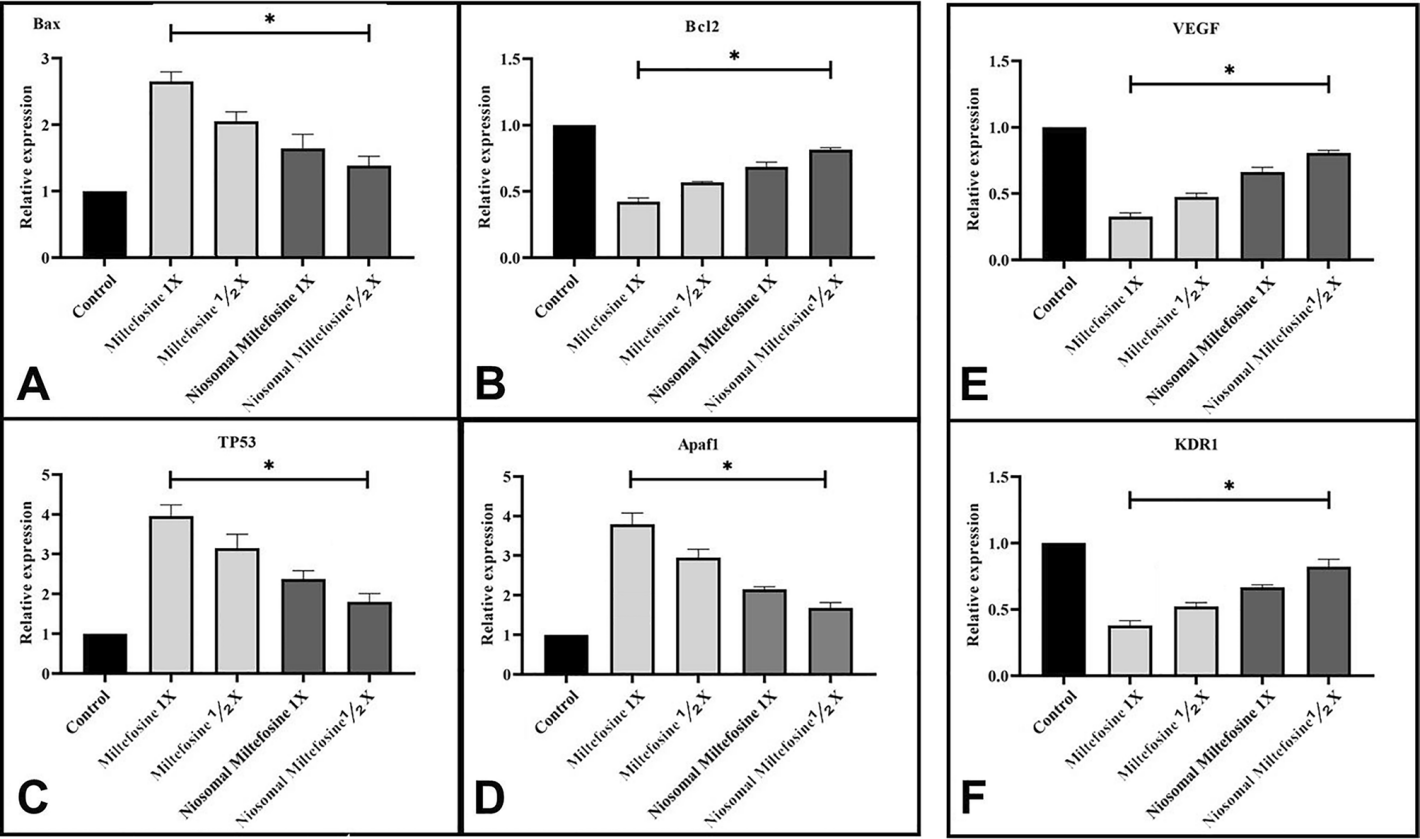
MIL and NMIL induced apoptotic moderator and angiogenesis genes in the chick’s extra-embryonic membrane vasculature. The expression level of the apoptotic mediator (**A**) Bax, (**B**) Bcl2, (**C**) TP53, and (**D**) Apaf 1. The angiogenesis genes (**E**) VEGF and (**F**) KDR the MIL and its NMIL treated embryos compared to controls. The expression profiles were standardized to GAPDH and HPRT and calibrated to controls (error bars demonstration standard mean error; *p < 0.05).

The results show that MIL alone significantly changed the gene expression of apoptosis mediators, including CASP8, APAf 1, Bcl-2, and BAX, compared to the control group (*P* < 0.05). Furthermore, the expression of apoptosis mediators in the NMIL group was lower than in the MIL group. Angiogenic mediators (KDR, VEGF, and AIF) in the MIL group were lower than those in the NMIL group, and both the MIL and NMIL groups differed significantly from the control group, as shown in Fig. 6.

#### IHC results

**Figure 7:**
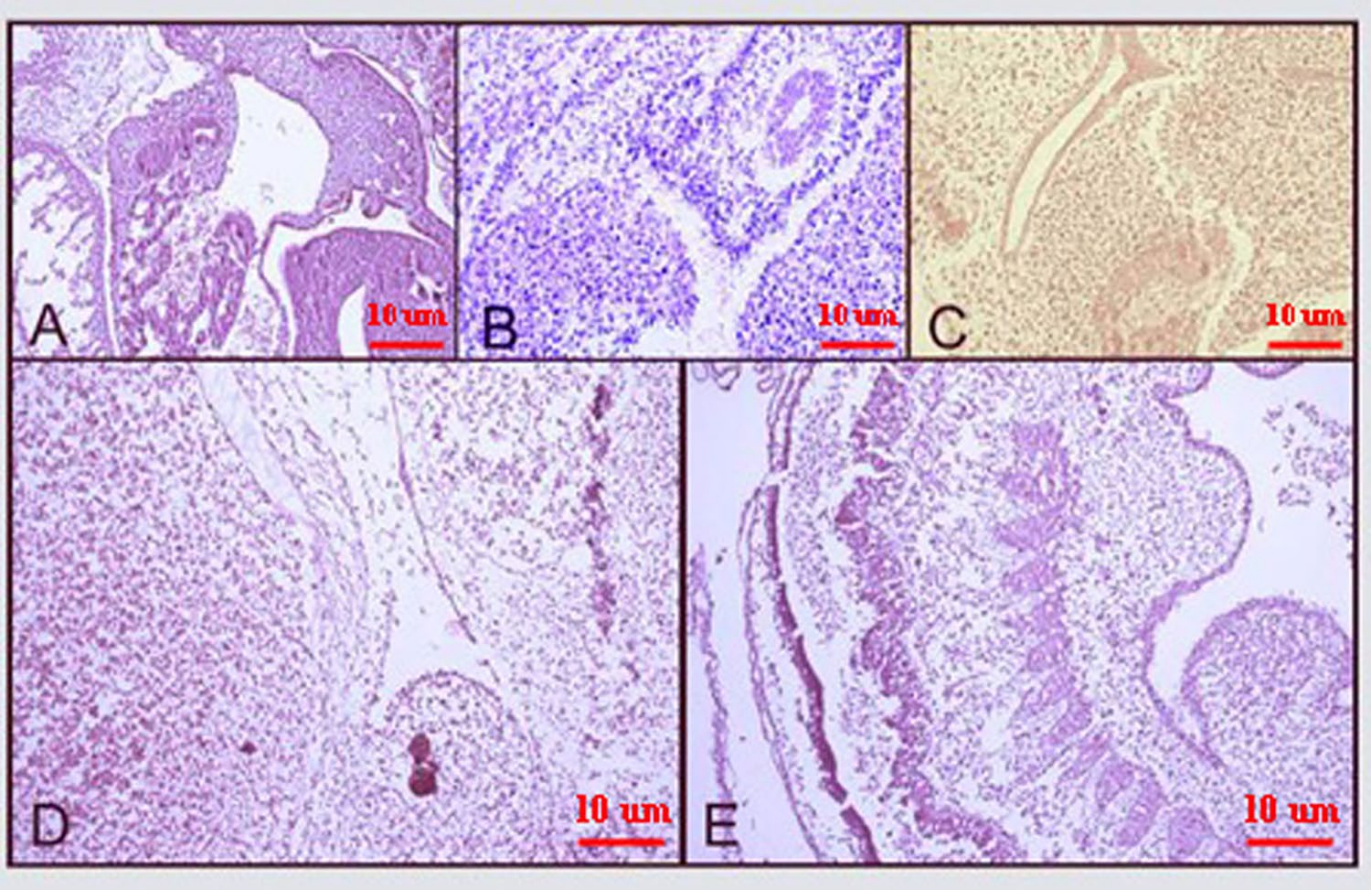
Microscopy images of H&E staining of chick embryo (**A**) control group, MIL (**B**) 1X, (**C**) 2X, (**D**) NMIL 1X, and (**E**) 2X.

**Figure 8:**
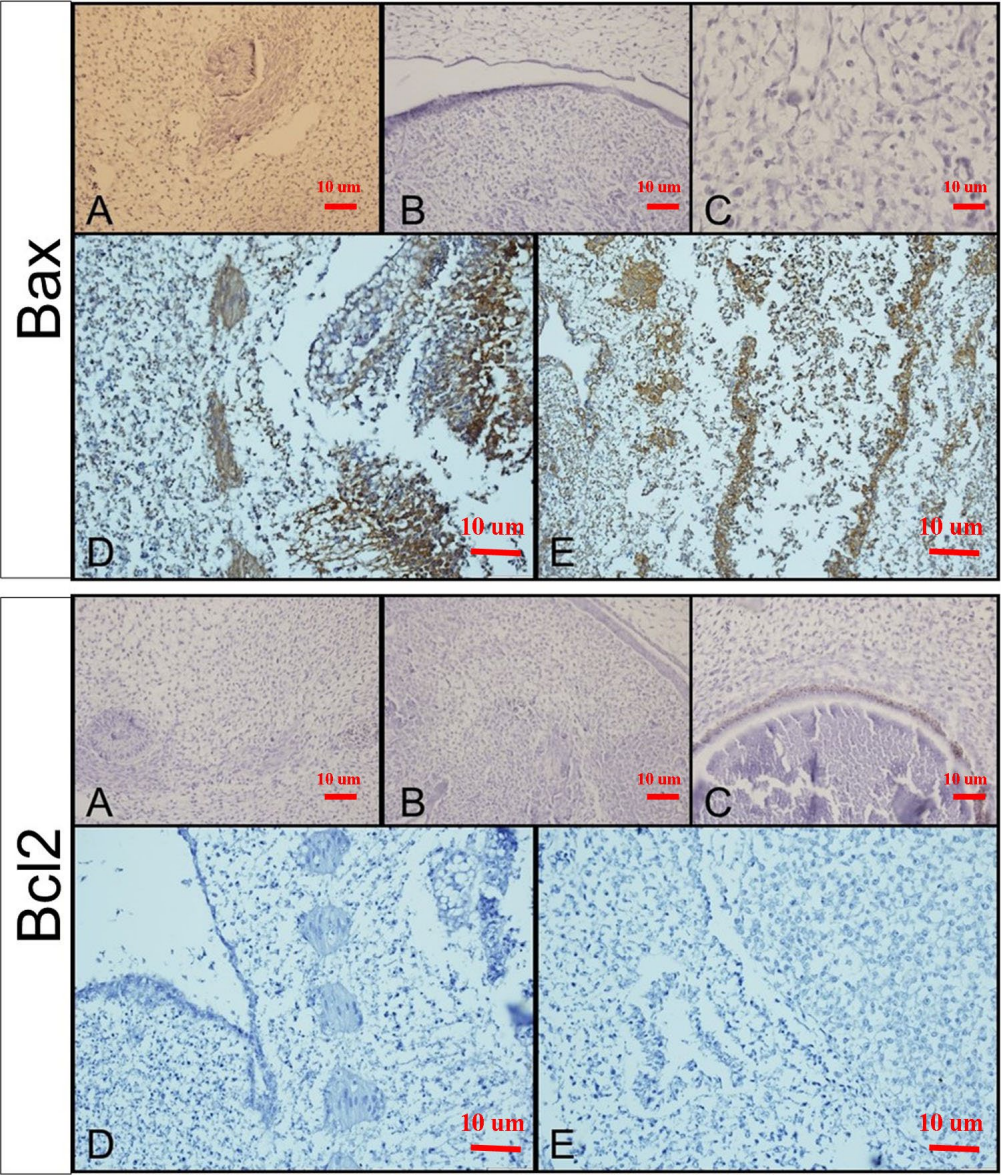
Microscopy images of IHC staining Bax (up) and Bcl 2 (down) of chick embryo (**A**) control group, MIL (**B**) 1X, (**C**) 2X, (**D**) NMIL 1X, and (**E**) 2X.

**Figure 9:**
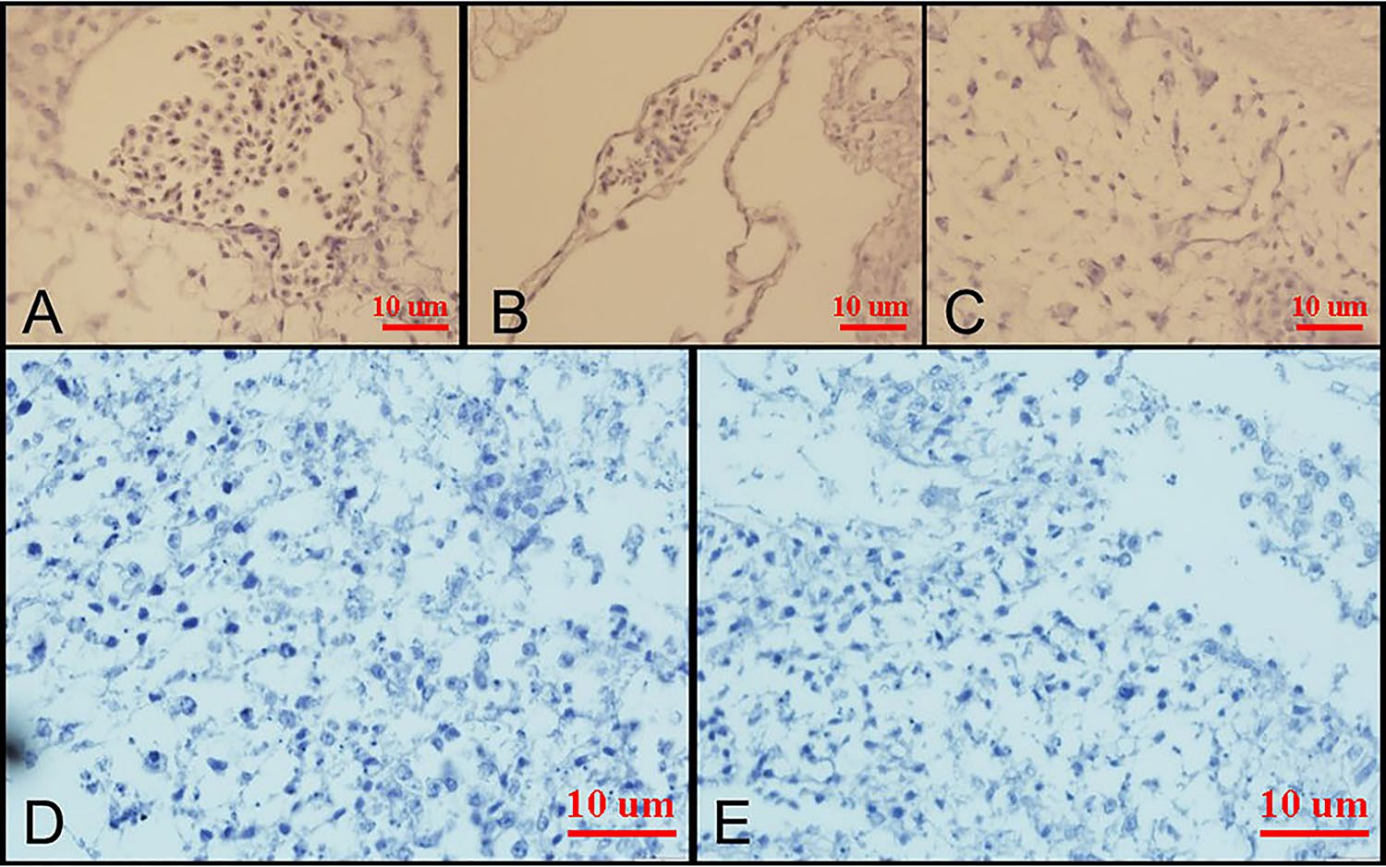
Microscopy images of IHC staining CD34 of chick embryo (**A**) control group, MIL (**B**) 1X, (**C**) 2X, (**D**) NMIL 1X, and (**E**) 2X.

The tissue changes in different therapeutic regimens upon organogenesis of the chicken embryo were evaluated by staining the tissue with Hematoxylin and Eosin. The results showed the presence of teratogenic effects, such as the absence of atrophic changes in the development of different organs. Additionally, degenerative or necrotic changes were observed in three other trilaminar embryonic germ cell lines, as shown in Figs. 7, 8 and 9.

## Discussion

Leishmaniasis is a significant global public health concern due to its high rate of illness and death caused by infections from the bite of female phlebotomine sandflies, which can spread to various mammalian species, including humans^47,48^. The major modes of transmission include blood transfusion and sharing of needles, and risk factors such as poverty, malnutrition, deforestation, and urbanization can increase the likelihood of infection. Clinical presentations of cutaneous leishmaniasis (CL) during pregnancy often manifest as exophytic lesions, and larger lesions are observed. Additionally, vertical transmission of visceral leishmaniasis (VL) can result in fetal loss if treatment is delayed or ineffective. Thus, treatment during pregnancy is crucial, taking into account the risk of miscarriage and prematurity^12,49,50^. However, the use of available therapies for leishmaniasis is limited by drug resistance, high toxicity, patient hospitalization, and high cost^51^.

Considering that no comprehensive study has been done on the effects of MIL on the embryo's health, this study was conducted to investigate the effects of this drug on the health of the chick embryo. For the first time, the effects of the Niosomal form as a nanoparticle on changes in the structure and the impact of the drug on changing the process of apoptosis and embryogenesis were investigated.

MIL is a phospholipid that acts as an antileishmanial agent. Its exact mode of action remains largely unknown, although several mechanisms have been proposed. One proposed mechanism is the inhibition of phosphatidylcholine synthesis, which is essential for forming Leishmania membranes. Another proposed mechanism is the inhibition of cytochrome c oxidase, which impairs the electron transport chain in the parasite's mitochondria. MIL has also been shown to disrupt the intracellular Ca^2+^ homeostasis, which can lead to cell death. Additionally, MIL has been found to disturb the Leishmania mitochondrion, leading to cellular dysfunction and death. Despite these proposed mechanisms, MIL's exact mode of action against Leishmania parasites is still not fully understood^52^.

Little information on the embryotoxicity properties of MIL has been reported^53^. As far as we know, Although previous studies revealed embryotoxic and fetotoxic efficacy of MIL in both rats and rabbits, no teratogenic effects were investigated^9^, and few studies were associated with the vascular apoptotic effect of NMIL of available therapies with their conventional forms by using the chick embryo model.

Because of its ease of use, ethical considerations, and lower cost, the chicken embryo is a suitable model for investigating the effects of drugs on fetal health [8,18]. This study has demonstrated the impact of MIL and NMIL on fetal growth, leading to disorders in fetal vascular development, increased apoptosis, and decreased vascular density during fetal development.

The present investigation on NMIL could be novel to contribute to its reduction of overall adverse effects while providing a potential strategy to modify the drug performance. As an indispensable part of modern medicine strategies, nanomedicine addresses challenges of infectious diseases such as leishmaniasis^54^. Niosomes and liposomes are practical examples of biocompatible and biodegradable nano-based drug delivery systems, which are composed of non-ionized surfactants and cholesterols, have been designed to avoid parasitic resistance, improve stability, enhance drug efficacy, and reduce the toxicity of their entrapped drug^55,56^.

The current investigation on NMIL is potentially groundbreaking as it may contribute to the reduction of overall adverse effects and provide a potential strategy to modify the drug's performance. Nanomedicine has become an indispensable part of modern medicine strategies and is used to address challenges such as infectious diseases, including leishmaniasis^54^. Niosomes and liposomes are practical examples of biocompatible and biodegradable nano-based drug delivery systems. These systems are composed of non-ionized surfactants and cholesterol and have been designed to avoid parasitic resistance, improve stability, enhance drug efficacy, and reduce the toxicity of the entrapped drug^55,56^. These delivery systems have shown great potential in reducing toxicity and improving the therapeutic effectiveness of drugs. They could potentially be applied to NMIL to enhance its safety profile and efficacy. Further studies are needed to explore this possibility and to optimize the formulation of these delivery systems for NMIL.

Our results demonstrated that the prepared niosomes had approximately a spherical shape and a diameter of less than 150 nm. The niosomes were composed of Span 60/Tween 60/Cholesterol compounds, and a high encapsulation rate of MIL was achieved (99.37%), which is sufficient for further investigations. The surface charge of nanoparticles positively affects the cellular interplay and nanoparticle uptake. Studies have shown that nanoparticles with negative or positive surface charges are more easily internalized than their uncharged counterparts^39^. However, nanoparticles with positive charges provide a more rapid cellular uptake than those with negative charges, but they are known to induce hemolytic effects and cytotoxicity in some organs^39,40^.

Bcl-2 and Bax family proteins are crucial in initiating cell apoptosis pathways. They activate this process by creating a cell signal within the mitochondria. The Bcl-2 family interacts with the carboxyl-terminal region of BAX^57^. The Bcl-2 family proteins regulate the mitochondrial outer membrane of the apoptosis process^42^. Molecular docking simulations were done for the dynamic genes of Bcl-2, BAX, Caspase-8, and VEGF-A to determine how MIL and NMIL could occupy active sites via various chemical bond interactions. Among these proteins, Bcl-2 and VEGF-A showed less binding affinity, while higher binding energy was observed for BAX and Caspase-8 towards NMIL compared to MIL.

This study shows that MIL and NMIL interact with the apoptotic and anti-apoptotic proteins BAX, Bcl-2 and Caspase-8. By contrast, the relative free total energy between the NMIL or MIL and apoptotic-regulator proteins provides significantly increased interactions, while the interactions decreased between BAX or Caspase-8 and Bcl2 proteins. Consequently, the NMIL was more effective than MIL alone.

VEGF-A is one of the most important agents for de novo blood vessel development^58^. The present investigation found that the binding affinity of MIL was higher than that of NMIL with VEGF-A. This study also observed the expression of apoptotic-control genes such as caspase domestic proteins (CASP), apoptotic protease activating factor (APAF), and apoptosis-inducing factor (TP53), as well as BAX and Bcl-2 genes. Bcl-2 prevents cell death, while BAX overexpression can accelerate cell death.

Recent investigations have shown that Bcl2 family proteins can interact with different genes, including Tp53, and suppress the transcription of anti-apoptotic genes such as Bcl2 and BclxL^45^. Interestingly, we observed that MIL and NMIL affected the Akt/PKB pathway, which led to a decrease in the expression of Bcl2 and an increase in the expressions of Bak and Bax genes. In the essential apoptosis pathway, changes in the permeability of the mitochondrial membrane to cytochrome c are regulated, mainly by pro-apoptotic mediators such as Bax and Bak.

In addition to its role in apoptosis, the release of VEGF-A can also activate other mechanisms, such as erythropoiesis, which involves the production of red blood cells. This is accomplished by binding the VEGF-A gene to its receptors on the endothelial cells, which leads to angiogenesis via the tyrosine kinase pathway^59,60^.

Based on our findings, the expression levels of some apoptotic-regulating genes, such as APAF1, were significantly increased in drug-exposed EEMs in a dose-dependent manner. The Tp53 gene is an essential gene involved in human cancers^61^. In this study, MIL and NMIL were found to significantly affect the expression of Tp53 and suppress the transcription of anti-apoptotic genes.

Our findings suggest that using NMIL instead of MIL alone in pregnant women may have fewer adverse effects on the fetus. To our knowledge, this is the first study to compare the toxicity of MIL alone and NMIL using a chick embryo model and to investigate their effects on various apoptotic-regulating genes.

## Conclusion

The results of this study showed that MIL can cause extensive changes in chicken embryos, indicating the toxic effects of this drug on the embryo's health during pregnancy. On the other hand, NMIL has a slower release due to its unique properties, which can reduce the drug's toxic effects and does not cause teratogenic changes like MIL. Therefore, administering this drug during pregnancy should be done with more caution. This study shows that MIL-loaded niosomes are a potent formulation for improving the pharmacokinetic and pharmacological properties of the entrapped drug. Due to the more effective interactions of NMIL of MIL with active regions, unique functional ability, and fewer side effects, it seems that NMIL is a good candidate for developing potential leishmaniasis management during pregnancy. However, further conclusive evidence is necessary to determine the teratogenic effect of MIL alone and the consequences of NMIL on the embryo throughout gestation in future clinical trials.

## Data Availability

The datasets used and analyzed during the current study are available from the corresponding author.
